# Aberrant Crossed Corticospinal Facilitation in Muscles Distant from a Spinal Cord Injury 

**DOI:** 10.1371/journal.pone.0076747

**Published:** 2013-10-17

**Authors:** Karen L. Bunday, Martin Oudega, Monica A. Perez

**Affiliations:** 1 Department of Physical Medicine and Rehabilitation, University of Pittsburgh, Pittsburgh, Pennsylvania, United States of America; 2 Systems Neuroscience Institute, University of Pittsburgh, Pittsburgh, Pennsylvania, United States of America; 3 Department of Bioengineering, University of Pittsburgh, Pittsburgh, Pennsylvania, United States of America; 4 Department of Neurobiology, University of Pittsburgh, Pittsburgh, Pennsylvania, United States of America; UMDNJ-Robert wood Johnson Medical School, United States of America

## Abstract

Crossed facilitatory interactions in the corticospinal pathway are impaired in humans with chronic incomplete spinal cord injury (SCI). The extent to which crossed facilitation is affected in muscles above and below the injury remains unknown. To address this question we tested 51 patients with neurological injuries between C2-T12 and 17 age-matched healthy controls. Using transcranial magnetic stimulation we elicited motor evoked potentials (MEPs) in the resting first dorsal interosseous, biceps brachii, and tibialis anterior muscles when the contralateral side remained at rest or performed 70% of maximal voluntary contraction (MVC) into index finger abduction, elbow flexion, and ankle dorsiflexion, respectively. By testing MEPs in muscles with motoneurons located at different spinal cord segments we were able to relate the neurological level of injury to be above, at, or below the location of the motoneurons of the muscle tested. We demonstrate that in patients the size of MEPs was increased to a similar extent as in controls in muscles above the injury during 70% of MVC compared to rest. MEPs remained unchanged in muscles at and within 5 segments below the injury during 70% of MVC compared to rest. However, in muscles beyond 5 segments below the injury the size of MEPs increased similar to controls and was aberrantly high, 2-fold above controls, in muscles distant (>15 segments) from the injury. These aberrantly large MEPs were accompanied by larger F-wave amplitudes compared to controls. Thus, our findings support the view that corticospinal degeneration does not spread rostral to the lesion, and highlights the potential of caudal regions distant from an injury to facilitate residual corticospinal output after SCI.

## Introduction

In healthy humans, the size of motor evoked potentials (MEPs) elicited by transcranial magnetic stimulation (TMS) in a resting arm muscle is increased by strong isometric voluntary contraction of the contralateral arm, a phenomenon referred to as crossed corticospinal facilitation [1–5]. This crossed facilitatory effect may favor interlimb coordination and motor performance [6–9]. In patients with chronic spinal cord injury (SCI), crossed corticospinal facilitation is impaired regardless of the magnitude of motor recovery [10]. However, the extent to which crossed corticospinal facilitation is affected in muscles above and below a SCI is unknown. 

Most injuries to the spinal cord are anatomically incomplete leaving some corticospinal axons intact. From damaged corticospinal axons, the caudal parts are gradually degenerated and ultimately cleared by macrophages (Wallerian degeneration; [11–13]), while the rostral parts show some retrograde degeneration [12–16]. Human magnetic resonance imaging studies have confirmed these different degenerative responses in the corticospinal tract below and above a SCI [17–19], but the extent of retrograde corticospinal degeneration after injury is still debated [17–19]. The reorganization in corticospinal neurons after SCI affects transmission in intact and injured corticospinal axons [20] and, therefore, may influence crossed corticospinal facilitation. In agreement, animal and human studies have shown that damage to the corticospinal tract results in impaired transmission in interhemispheric interactions between primary motor cortices [10,21], which is a mechanism contributing to crossed corticospinal facilitation [4,10]. After SCI, ipsilateral and contralateral corticospinal projections sprout extensively above and below the lesion [22,23] and across the midline [24]. These crossed corticospinal projections are thought to affect crossed corticospinal facilitation [25]. Bareyre and colleagues [26] demonstrated that corticospinal sprouts that contacted neurons with short projections are typically lost, while sprouts that contact neurons with longer projections that extend to segments distant from the lesion remain. The reorganization present at greater distances below the injury contributes to further enhance physiological and motor outcomes after SCI [27]. Therefore, we hypothesized that crossed corticospinal facilitation will be present in muscles above the injury and further increased at a greater distances below the injury. 

To test our hypothesis, we examined 51 individuals with neurological injuries between C2-T12 spinal cord segments and tested crossed facilitation in muscles which motoneurons were located above, at, and at a distance from the lesion and 17 age-matched healthy controls. We used TMS to elicit MEPs in a resting hand, arm, or foot muscle when the contralateral side remained at rest or performed 70% of maximal voluntary contraction (MVC). 

## Methods

### Ethics Statement

The study was performed in accordance with the Declaration of Helsinki. All subjects gave their informed consent to the experimental procedures, which was approved by the local ethics committee at the University of Pittsburgh. Patients were recruited from the Department of Physical Medicine and Rehabilitation research registry at the University of Pittsburgh. 

### Subjects

Fifty-one patients with chronic (≥ 1 year) SCI and 17 age-matched healthy controls (SCI: mean age=46.7±13.2 yr, 7 female, Tables 1 and 2; age-matched healthy controls: mean age=40.8±17.2 yr, 5 male; p=0.17) participated in the study. All patients had the neurological level of injury between C2-T12 spinal cord segments (31 patients had a cervical SCI, Table 1; 20 patients had a thoracic SCI, Table 2). Nineteen (7 cervical SCI, 12 thoracic SCI) out of 51 patients were categorized using the American Spinal Injury Association impairment scale (AIS) based on the International Standard for Neurological Classification of SCI as AIS A (complete injury) due to the lack of sacral sparing, and were able to elicit voluntary force with hand and arm muscles. The other 32 patients were classified as incomplete AIS B (2 cervical SCI), C (3 cervical SCI, 1 thoracic SCI) and D (19 cervical SCI, 7 thoracic SCI). To examine crossed facilitation in the corticospinal pathway in muscles located above, at, and below the injury site we used TMS to elicit MEPs in the resting first dorsal interosseous (FDI, motoneurons located at C8-T1), biceps brachii (BB, motoneurons located at C5-C6), and tibialis anterior (TA, motoneurons located at L4-L5) muscles when the contralateral side remained at rest or performed 70% of MVC into index finger abduction, elbow flexion, and ankle dorsiflexion, respectively. By testing MEPs in muscles with motoneurons located at different spinal cord segments we were able to relate the neurological level of injury to be above, at, or below the location of the motoneurons for the muscle tested. With injuries at the same location of the motoneurons of the muscles tested, measurements will be referred to as “at the injury site”. Then, measurements were at the injury site for patients with lesions between C5-C6 segments for BB and for patients with injuries between C8-T1 segments for FDI. With injuries above the location of the motoneurons for the muscles tested, measurements will be referred to as “below the injury site”. This group includes patients with injuries only between C2 and C7. Because no differences were found between MEP data gathered at rest and during 70% of MVC from SCI groups C2, C3, and C4 and between C5, C6, and C7 the results of these groups were pooled together. In patients with injuries between C2-C4, MEPs in the BB were located ~1–4 segments below the injury, MEPs in the FDI were located ~4–7 segments below the injury, and MEPs in the TA were located ~20–23 segments below the injury. In patients with injuries between C5-C7, MEPs in the TA were located ~17–20 segments below the injury. With injuries below the location of the motoneurons for the muscles tested, measurements will be referred to as “above the injury site”. This group includes patients with lesions between T3-T12. Because no differences were found between MEP data gathered at rest and during 70% of MVC from SCI groups T3, T4, and T5 and between T6-T12 the results of these groups were pooled together. In this group, with an injury between T3-T5, MEPs in the FDI were ~2–5 segments above the injury, while MEPs in the BB were ~6–8 segments above the injury. With injuries between T6-T12, MEPs in the FDI were ~6–12 segments above the injury and MEPs in the BB were ~8–15 segments above the injury. Group comparisons (“at the injury site”, “below the injury site”, and “above the injury site”) showed that the time since injury was similar across groups (F_(4,50)_=1.4, p=0.26).

**Table 1:** Cervical spinal cord injury participants.

	Patient	Age (yrs)	Gender	ASIA Score	Level	Aetiology	Time (yrs)	FDI MVC (N)	BB MVC (N)
**C2-C4**	1	56	M	D	C_2_	T	3	14.8	22.2
	2	62	M	D	C_3_	T	2	17.8	119.9
	3	46	M	D	C_4_	T	9	2.4	54.4
	4	63	M	D	C_4_	T	6	16.2	99.9
	5	58	M	C	C_4_	NT	4	8.4	154.4
	6	66	M	D	C_4_	T	3	14.6	73.9
	7	57	F	D	C_4_	T	5	7.6	94.1
	8	41	M	D	C_4_	T	26	n/t	n/t
	9	54	M	D	C_4_	T	1	n/t	n/t
**C5-C6**	10	62	M	D	C_5_	T	5	19.1	145.2
	11	50	M	D	C_5_	T	1	14.4	136.1
	12	38	M	D	C_5_	T	1	11.1	78.3
	13	57	M	D	C_5_	T	5	13.9	130.1
	14	61	M	A	C_5_	T	10	n/a	12.5
	15	39	M	A	C_5_	T	19	n/a	135.6
	16	27	M	A	C_6_	T	13	n/a	179.3
	17	30	M	A	C_6_	T	6	21.4	147.5
	18	26	M	D	C_6_	T	11	1.5	195.6
**C7**	19	32	M	D	C_7_	T	9	9.2	93.2
	20	59	F	D	C_7_	T	13	7	118.6
	21	45	M	D	C_7_	T	6	4.4	144.6
	22	42	F	C	C_7_	T	20	11.9	81.7
	23	58	M	D	C_7_	T	2	16.7	113.9
	24	47	M	D	C_7_	T	13	n/t	n/t
	25	53	M	D	C_7_	T	3	7.7	251.3
**C8-T1**	26	56	M	A	C_8_	T	1	19	150.2
	27	45	M	C	C_8_	T	9	24.9	168.5
	28	35	M	A	C_8_	T	17	1.3	138.5
	29	42	M	A	C_8_	T	13	1.5	187.7
	30	23	M	B	C_8_	T	7	6.9	88.5
	31	53	M	B	T_1_	T	12	27	239.4

M = Male, F = Female, ASIA = American Spinal Injury Association impairment scale, T = Traumatic, NT = Non-traumatic, MVC = Maximum voluntary contraction, N = Newtons, n/a = not applicable, patient completed elbow flexion only, n/t = not tested, patient completed dorsiflexion only.

**Table 2:** Thoracic spinal cord injury participants.

	Patient	Age (yrs)	Gender	ASIA Score	Level	Aetiology	Time (yrs)	FDI MVC (N)	BB MVC (N)
**T3-T5**	1	48	M	D	T_3_	T	21	21.8	177.9
	2	38	M	A	T_4_	T	15	47.7	266.1
	3	31	M	A	T_5_	T	4	17.6	90.1
	4	38	F	D	T_5_	T	1	7.5	64.8
	5	26	M	A	T_5_	T	22	19.9	111.6
	6	43	F	A	T_5_	T	27	20.1	95.4
**T6-T12**	7	44	M	A	T_6_	T	16	26.6	141.1
	8	61	M	C	T_6_	NT	3	17.1	83.5
	9	58	F	D	T_7_	T	16	11.3	80.1
	10	57	M	D	T_7_	T	3	43.7	284.2
	11	57	M	A	T_7_	T	26	12.5	130
	12	55	M	A	T_8_	T	11	11.2	303
	13	31	M	A	T_8_	T	12	24.4	80
	14	31	M	A	T_9_	T	9	24.9	154.3
	15	57	M	D	T_10_	T	7	22.4	254.2
	16	26	M	A	T_10_	T	4	32	148.4
	17	23	M	A	T_10_	T	3	33.6	196.4
	18	52	F	D	T_11_	T	7	16	148.5
	19	68	M	A	T_11_	T	20	8.7	132.6
	20	67	M	D	T_12_	T	5	14.7	123.8

M = Male, F = Female, ASIA = American Spinal Injury Association impairment scale, T = Traumatic, NT = Non-traumatic, MVC = Maximum voluntary contraction, N = Newtons.

### Recordings

Electromyography (EMG) was recorded bilaterally from the FDI, BB, and TA by surface electrodes secured to the skin over the belly of each muscle (Ag–AgCl, 10 mm diameter). The signals were amplified, filtered (20–1000 Hz), and sampled at 2 kHz for off-line analysis (CED 1401 with Signal software, Cambridge Electronic Design, Cambridge, UK). Forces exerted at the proximal interphalangeal joint of the index finger, at the elbow, and at the ankle joint were measured by load cells (Honeywell, Ltd., range ± 498.1 N, voltage ± 5 V, high-sensitivity transducer 0.045 V/N). Force was sampled at 200 Hz and stored on a computer for off-line analysis.

### Experimental Setup

**Figure 1:**
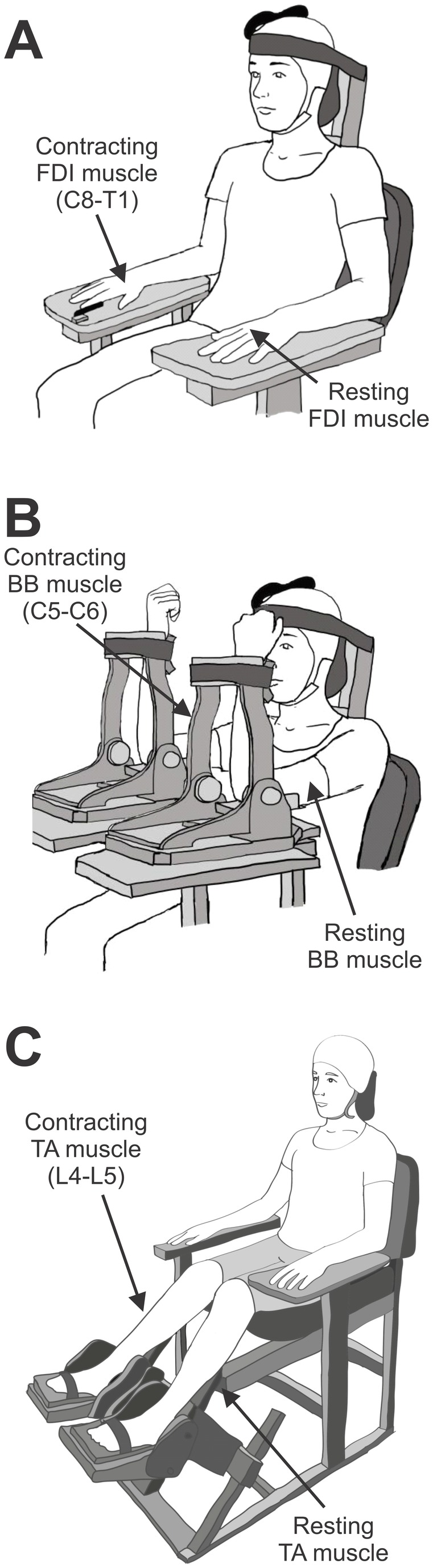
Experimental Set-up. Diagrams show the posture of the hand, elbow, and foot used during testing. Three motor tasks were randomly tested. In task 1 (A), subjects were seated in an armchair with both arms flexed at the elbow by 90° with the forearm pronated and the wrist and forearm restrained by straps. In this task, crossed motor evoked potential (MEP) facilitation was examined in the first dorsal interosseous (FD, motoneurons located at C8-T1) muscle. In task 2 (B), subjects were seated in an armchair with both shoulders and elbows flexed by 90°. In this task crossed MEP facilitation in the biceps brachii (BB, motoneurons located at C5-C6) muscle was tested. In task 3 (C), subjects were seated in an armchair with both feet attached to a footplate. Here crossed MEP facilitation was tested in the tibialis anterior (TA, motoneurons located at L4-L5) muscle. During all tasks subjects were at rest while the contralateral side remained at rest or performed 70% of MVC into index finger abduction (A), elbow flexion (B), and ankle dorsiflexion (C).

Subjects were randomly tested in 3 motor tasks. In one task (Figure 1A), participants were seated in an armchair with both arms flexed at the elbow by 90° with the forearm pronated and the wrist and forearm restrained by straps. In this position, index fingers were attached to custom two-axis load cells (Honeywell, Ltd.), which measures finger abduction force. In another task (Figure 1B), testing was completed with both shoulders and elbows flexed by 90°. A custom device was used to maintain the position of each arm with two-axis load cells (Honeywell, Ltd.) attached to measure elbow flexion forces. In a third task (Figure 1C), the arm was maintained in the same position as described above, while the feet were attached to a footplate connected to custom two-axis load cells (Honeywell, Ltd) to measure dorsiflexion forces. At the start of the experiment, subjects performed 3 brief MVCs (3-5 s) into index finger abduction, elbow flexion, and ankle dorsiflexion separated by 30 s. The maximal forces were used to set targets for subsequent submaximal contractions. During testing, subjects were at rest while the contralateral side remained at rest or performed 70% of MVC into index finger abduction, elbow flexion, and ankle dorsiflexion. Patients performed contractions with the least affected limb and healthy controls used the right dominant side. In patients in whom we were unable to elicited MEPs in the more affected limb, the least affected limb was tested. Custom software (LabView, California) was written to acquire signals from the load cell and to display visual feedback corresponding to rest and MVC levels in real time. During testing subjects were instructed to move a cursor to a target box presented on a computer monitor by performing index finger abduction, elbow flexion, and ankle dorsiflexion. A familiarization trial was completed at the beginning of each experiment to ensure that subjects were able to complete the task. EMG from the resting muscles was displayed continuously on an oscilloscope and verbal feedback was provided to the subjects to assure that physiological measurements in the FDI, BB, and TA were acquired at rest at all times. To ensure that the same background activity was present in both conditions, trials in which mean rectified EMG activity in the resting muscles exceeded 2 SD of the mean resting EMG, measured 100 ms before the stimulus artifact, were excluded from further analysis. A total of 8.9% trials were excluded from the analysis [4,10]. 

### TMS

Transcranial magnetic stimuli were delivered from a Magstim 200 stimulator (Magstim Company) through a figure-eight coil (type number SP15560) or a double cone coil (type number 9902-00) with a monophasic current waveform. TMS was delivered to the optimal scalp position for activation of the left or right FDI, BB, and TA muscles. To identify the optimal scalp position for both the FDI and BB the coil was held tangential to the scalp with the handle pointing backward and 45° away from the midline. With this coil position the current in the coil flowed in a posterior-anterior direction and probably produced D and early I wave activation of corticospinal neurons [28]. For activation of the TA muscle, the center of the coil was positioned over the leg representation of the motor cortex 1 cm to the left or right from the vertex with the handle pointing backwards [29]. The TMS coil was held to the head of the subject with a custom coil holder, while the head was firmly secured to a headrest by straps to limit head movements. TMS measurements included MEPs, resting motor threshold (RMT), and maximal MEP size (MEP-max).

### MEPs

RMT for all muscles tested was defined as the minimal stimulus intensity required to induce MEPs greater than 50 μV peak-to-peak amplitude in at least 3/5 consecutive trials in the relaxed muscle ([30]; Table 3). The MEP-max was defined in all participants at rest by increasing stimulus intensities in 5% steps of maximal device output until the MEP amplitude did not show additional increases (Table 3). TMS intensity (expressed as % of the RMT) used to elicit MEPs around 50% of the MEP-max was similar across groups (Table 3). Single TMS pulses were delivered at 4 s intervals in sets of 3 and separated by resting periods as needed. Thirty MEPs were averaged in each condition. TMS pulses were given when subjects were at rest or performed 70% of MVC during index finger abduction, elbow flexion, and ankle dorsiflexion in a randomized order. 

**Table 3:** Stimulation parameters, MEP-max and M-max (mean±SD).

		Healthy Controls	C2-C4	C5-C7	C8-T1	T3-T5	T6-T12	p-values
RMT (% MSO)	FDI	46.02±8.8%	75.1±17.4%^*^	61.9±20.0%	48.1±3.9%	50.7±11.1%	54.1±16.0%	p<0.01
	BB	62.9±12.1%	84.4±16.9%	71.3±21.9%	75.3±21.5%	66.0±2.0%	68.1.±14.0%	p=0.19
	TA	62.9±12.1%	69.8±11.3%	82.8±15.9%^*^				p<0.05
MEP-max	FDI	5.0±2.7 mV	0.8±0.3 mV^*^	2.1±2.8 mV	2.6±2.2 mV	4.1±3.6 mV	5.4±3.6 mV	p<0.01
	BB	0.8±0.6 mV	0.4±0.2 mV	1.2±1.3 mV	0.8±0.7 mV	0.7±0.7 mV	0.7±0.6 mV	p=0.60
	TA	1.7±1.0 mV	0.7±0.4 mV	0.6±0.5 mV				p<0.05
Stimulation	FDI	124.9±12.2%	122.1±3.3%	119.2±8.8%	121.0±3.3%	121.8±4.2%	122.6±8.8%	p=0.32
Intensity (% RMT)	BB	134.9±20.9%	117.4±4.3%	119.2±6.0%	114.5±7.4%	122.3±1.5%	120.3±7.2%	p=0.08
	TA	119.8±19.3%	125.9±4.4%	121.8±1.1%				p=0.76
M-max	FDI	18.7±4.5 mV	13.4±8.4 mV	12.7±6.8 mV	10.1±7.1 mV	16.9±6.5 mV	19.6±5.8 mV	p<0.05
	BB	7.1±5.1 mV	7.1±5.2 mV	10.8±4.5 mV	14.5±3.6 mV	8.6±6.6 mV	5.4±2.7 mV	p=0.10
	TA	6.5±2.3 mV	5.3±3.9 mV	3.9±1.4 mV				p=0.22

RMT = resting motor threshold, MSO = maximum stimulator output, FDI = first dorsal interosseous, BB = biceps brachii, TA = tibilias anterior. P values represent one way ANOVA tests performed on RMT, MEP-max, stimulation intensity (% of RMT) and M-max, asterisks (*) denotes p<0.05 compared to healthy controls.

### F-Wave

Motoneuronal excitability could also contribute to the observed changes in MEP size. We examined the excitability of TA motoneurons by assessing the amplitude and persistence of F-waves in the resting TA during contralateral dorsiflexion at 70% of MVC. F-waves reflect backfiring of a small number of motoneurons which are reactivated by antidromic impulses following supramaximal stimulation of a peripheral nerve [31]. F-waves were measured by stimulating the common peronneal nerve behind the head of the fibula (200 µs pulse duration, DS7A, Digitimer). The stimuli were delivered at 1Hz at an intensity of 120% of the maximal motor response (M-max). The M-max reflects activation of all motoneurons and it was measured in the FDI, BB, and TA muscles by using supra-maximum stimulus intensity to the ulnar nerve, brachial plexus at erb’s point, and the common peroneal nerve was used to measure the M-max (Table 3), respectively. For each trial, we quantified the peak-to-peak amplitude (expressed relative to the M-max) and F-wave persistence (number of F-waves present in each set). If the F-wave was not present an amplitude of zero was included in the mean [10]. A total of 60 F-waves were averaged in the resting TA muscle when the contralateral TA remained at rest or performed 70% of MVC with ankle dorsiflexors. 

### Data Analysis

Normal distribution was tested by the Shapiro-Wilk's test and homogeneity of variances by the Levene’s test of equality and Mauchly’s test of sphericity. When sphericity could not be assumed, the Greenhouse-Geisser correction statistic was used. Repeated-measures ANOVAs were performed to determine the effect of task (rest and 70% of MVC) and group (healthy controls and SCI) on MEP size and mean rectified EMG for muscles located “at the injury site”, “below the injury site”, and “above the injury site”. Additional repeated-measures ANOVAs were performed to determine the effect of task (rest and 70% of MVC), muscle (BB, FDI, and TA) and group (healthy controls and SCI) on MEP size for muscles located “below the injury site”. Bonferroni *post hoc* tests were used to test for significant comparisons. Paired t-tests comparing rest and 70% of MVC were completed for BB, FDI, and TA MEP size on each group separately. One-way ANOVA was performed to determine the effects of force, RMT, stimulator output intensity (% of RMT), MEP-max, and M-max during the tasks tested. Significance was set at p<0.05. Group data are presented as the means ± SD in the text. 

## Results

### MVCs

Participants were able to exert MVC isometric forces into elbow flexion (healthy controls=138.3±48.4 N, cervical SCI=127.0±56.1 N, and thoracic SCI=153.3±72.4 N, p=0.35), index finger abduction (healthy controls=17.9±6.6 N, cervical SCI=12.0±7.3 N, and thoracic SCI=21.7±6.5 N, p=0.001), and dorsiflexion (healthy controls=161.6±57.8 N, cervical SCI=148.3±47.1 N, p=0.57). Only three patients with cervical SCI were unable to generate index finger voluntary force (Table 1, patients 14, 15, and 16) and were only tested for the elbow flexion. 

### MEPs at the injury site

**Figure 2:**
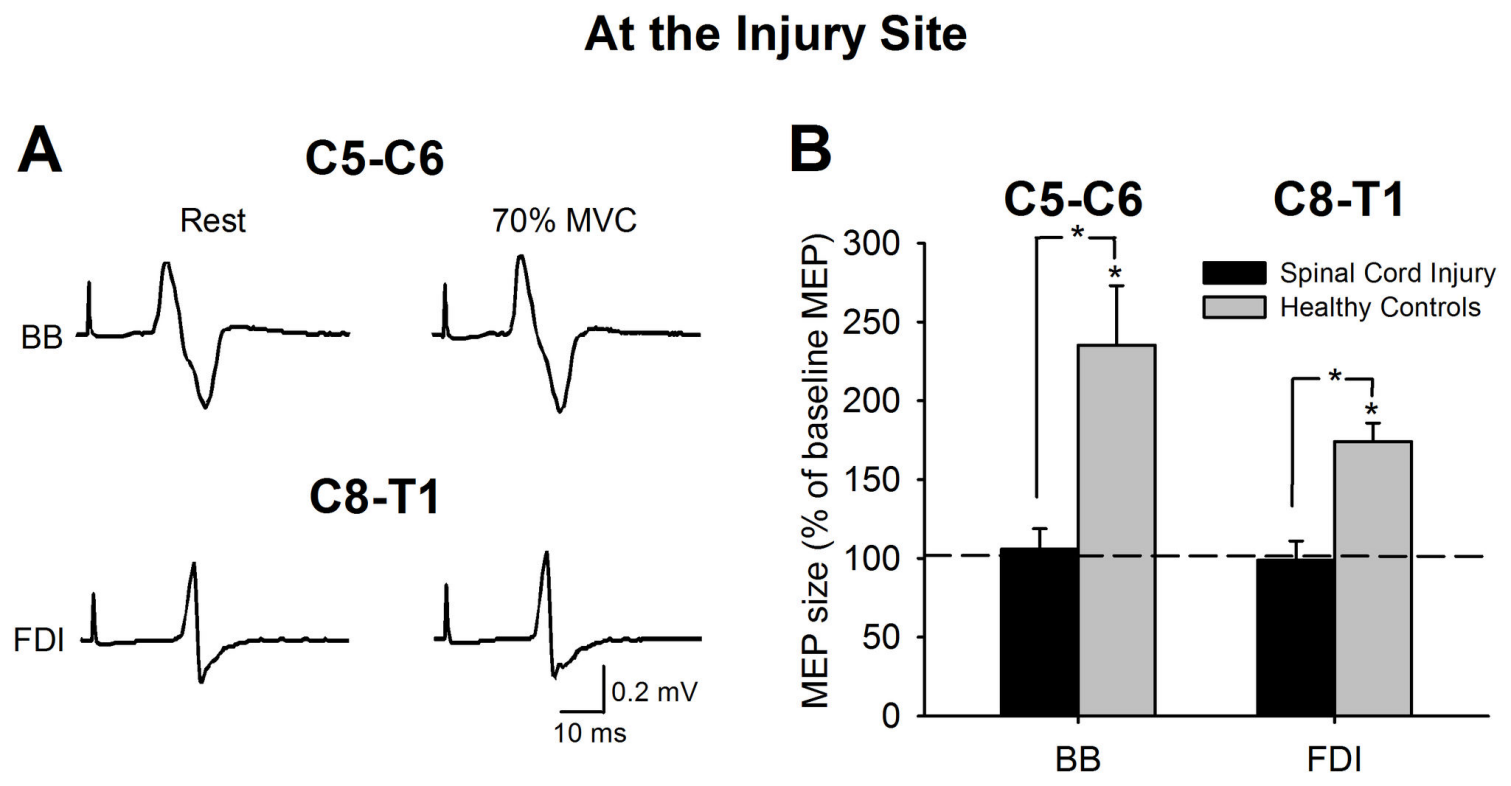
MEPs at the injury site. (A) MEPs recorded from the resting BB and FDI of representative patients with a C5-C6 and C8-T1 SCI, while the other side remained at rest or performed 70% of elbow flexion or index finger abduction. Group data (B, healthy controls, n=17; C5-C6, n=9, C8-T1, n=5). The abscissa shows the muscle tested (BB and FDI). The ordinate shows the size of BB and FDI MEPs as a % of the baseline BB and FDI MEPs. Note the increase in BB and FDI MEP size during contralateral index finger abduction and elbow flexion in healthy controls, but not in patients with C5-C6 and C8-T1 SCI. Error bars indicate SEs. *p*<*0.05.

Figure 2 illustrates MEPs in representative patients with cervical SCI recorded from the resting BB and FDI during the elbow flexion and index finger abduction. Note the increase in MEP size in both muscles during 70% of MVC in the healthy controls (see gray bars in the graph), but not in the BB in the patients with C5-C6 injuries and in the FDI in the patients with C8-T1 injuries. 

Repeated-measures ANOVA showed a significant effect of group (F_(1,14)_=5.0, p=0.04), task (F_(1,14)_=6.0, p=0.03), and in their interaction (F_(1,14)_=5.0, p=0.04) on BB MEPs size. We found that MEP size in the BB muscle was increased in healthy controls (235.1±125.4%, p=0.005; Figure 2B), but not in patients with C5-C6 injuries (105.8±29.0%, p=0.68; Figure 2B) during 70% of MVC compared to rest. A significant effect of group (F_(1,21)_=12.6, p=0.002), task (F_(1,21)_=11.8, p=0.002), and in their interaction (F_(1,21)_=12.6, p=0.002) was also found on FDI MEPs size. Here, we show that FDI MEP size increased in healthy controls (174.1±48.3%, p<0.001; Figure 2B), but not in patients with C8-T1 injuries (98.9±30.0%, p=0.93; Figure 2B) during 70% of MVC compared to rest. Mean background rectified EMG activity in the resting FDI and BB remained similar across conditions for all groups (F_(1,35)_=2.8, p=0.11). Overall, our results demonstrate that during strong unilateral isometric voluntary contractions healthy controls increase the size on MEPs in the contralateral resting homonymous muscle while in patients with chronic cervical SCI, MEPs were not facilitated when the motoneurons of the muscle tested were located at the injury site. 

### MEPs below the injury site

**Figure 3:**
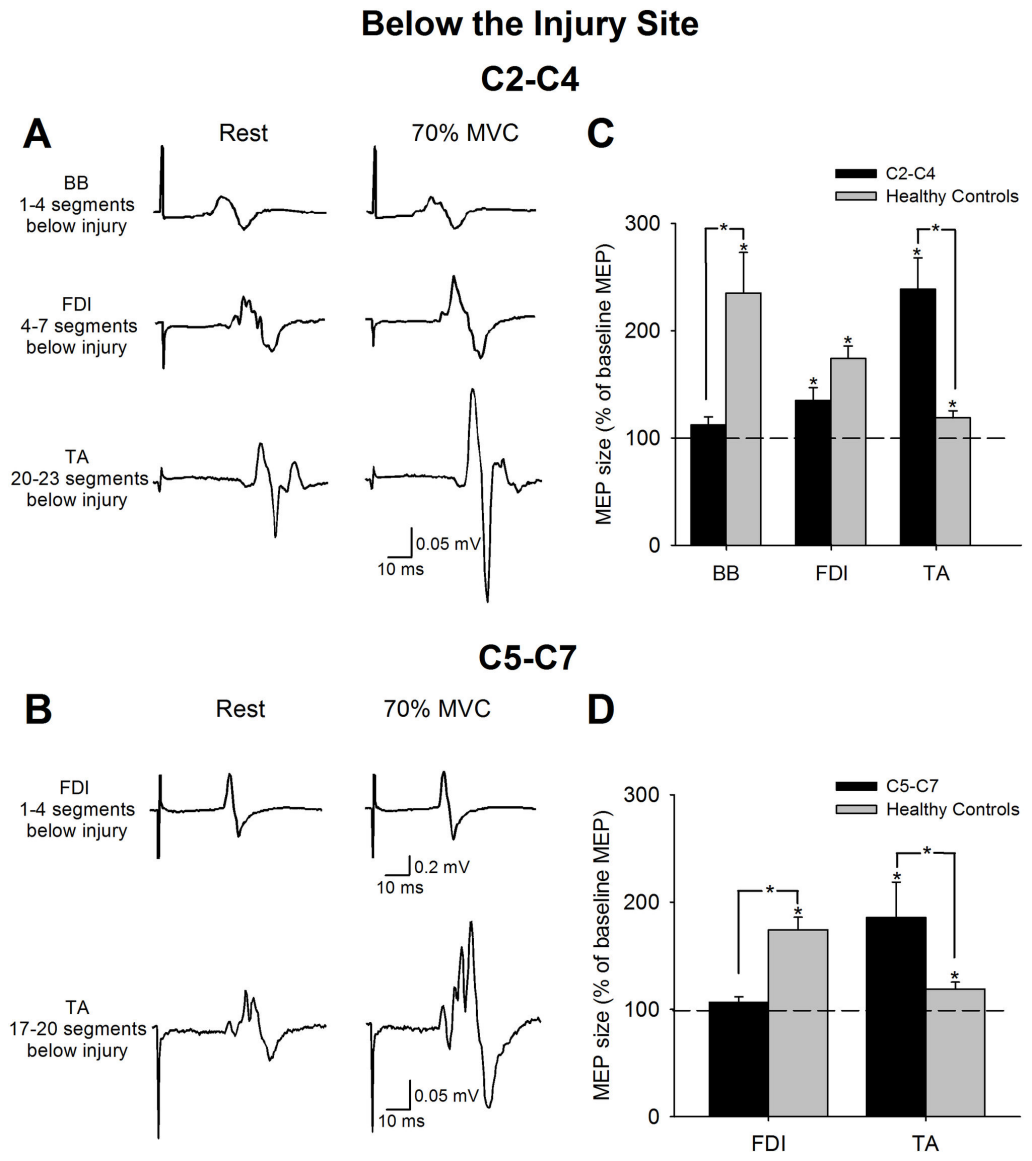
MEPs below the injury site. MEPs recorded from the resting BB, FDI, and TA of representative patients with a C2-C4 (A) and C5-C7 injury (B), while the other side remained at rest or performed 70% of index finger abduction, elbow flexion, or ankle dorsiflexion. Group data (C, healthy controls, n=17 and C2-C4, n=7; D, healthy controls, n=16 and C5-T7, n=11). The abscissa shows the muscle tested (BB, FDI, and TA). The ordinate shows the size of BB, FDI, and TA MEPs as a % of the baseline BB, FDI and TA MEPs. Note the increase in FDI and TA MEP size during contralateral index finger abduction and elbow flexion, but not in BB MEPs during elbow flexion in patients with C2-C4 injuries. Interestingly, TA MEPs size was increased in both groups of patients more than in healthy controls. Error bars indicate SEs. *p*<*0.05.

Figure 3 illustrates MEPs from representative patients with cervical SCI recorded from the resting BB, FDI, and TA during contralateral index elbow flexion, finger abduction, and ankle dorsiflexion (70% of MVC). Note that in patients with injuries between C2-C4, MEP size in the FDI and TA muscles increased, but remained unchanged in the BB during 70% of MVC compared to rest. Also, patients with injuries between C5-C7, FDI MEP size showed no changes in the FDI, but increased MEP size in TA during 70% of MVC compared to rest. 

During comparisons between C2-C4 patients and healthy controls, repeated-measures ANOVA showed a significant effect of task (F_(1,16)_=9.3, p=0.008), group (F_(1,16)_=6.5, p=0.02), and in their interaction (F_(1,16)_=6.5, p=0.02) on BB MEPs size. Here, we found that BB MEP size was significantly increased in healthy controls (235.1±125.4%, p=0.005; Fig. 3AC) but not in patients (112.0±20.3%, p=0.17; Fig. 3AC) during 70% of MVC compared to rest. This result indicated that MEP crossed facilitation was impaired when the motoneurons of the muscle tested are located within 5 segments below the injury site. Additionally, a significant effect of task (F_(1,22)_=29.8, p<0.001), but not group (F_(1,22)_=3.8, p=0.06), nor in their interaction (F_(1,16)_=6.5, p=0.02) was found on FDI MEPs size. We show that healthy controls (174.1±48.3%, p<0.001; Fig. 3AC) and patients (135.0±31.9%, p=0.02; Fig. 3AC) significantly increased FDI MEP size during 70% of MVC compared to rest, suggesting that MEP crossed corticospinal facilitation was preserved when the motoneurons of the muscle tested were located beyond 5 segments below the injury site. 

To further study the effects of crossed facilitation in muscles located at greater distances below the injury we tested the TA in the same group of C2-C4 patients and in healthy controls. Repeated-measures ANOVA showed a significant effect of task (F_(1,13)_=53.7, p<0.001), group (F_(1,13)_=30.42.7, p<0.001), and in their interaction (F_(1,13)_=30.42.7, p<0.001) on TA MEPs size. Similar to the results in the FDI muscle, we found that MEPs size in the TA were increased in healthy controls (118.7±20.9%, p=0.02; Fig. 3AC) and in patients (233.0 ±60.5%, p=0.008; Fig. 3AC) during 70% of MVC compared to rest. However, in contrast to the results in the FDI muscle, we found that the increases in TA MEP size were larger in patients compared to healthy controls (p<0.001). 

We also compared MEP crossed facilitation between C5-C7 patients and healthy controls. Repeated- measures ANOVA showed a significant effect of task (F_(1,27)_=29.5, p<0.001), group (F_(1,27)_=20.7, p<0.001), and in their interaction (F_(1,27)_=20.7, p<0.001) on FDI MEPs size. We found that healthy controls (174.1±48.3%, p<0.001; Fig. 3BD) but not patients (106.5±20.0%, p=0.28; Fig. 3BD) increased the size of MEPs in the FDI muscle during 70% of MVC compared to rest. This result indicates that in patients with C5-C7 injuries, as in patients with C2-C4 injuries, MEP crossed corticospinal facilitation is disrupted when the motoneurons of the muscle tested were located within 5 segments below the injury site. We also found a significant effect of task (F_(1,14)_=15.6, p=0.001), group (F_(1,14)_=6.4, p=0.02), and in their interaction (F_(1,14)_=6.4, p=0.02) on TA MEPs size. In contrast to FDI MEPs, both patients (185.5±80.9%, p=0.04; Fig. 3BD) and healthy controls (118.7±20.9%, p=0.02; Fig. 3BD) showed an increase in TA MEP size during 70% of MVC compared to rest. Importantly, we found that the increases in TA MEP size were 2-fold above healthy controls in C5-C7 patients (p<0.001). 

To further investigate the mechanism involved in the increased TA MEP size in C2-C7 patients compared to healthy controls we tested F-waves in the TA at rest and during 70% of MVC in both groups. The M-max was similar across groups (healthy controls=6.5±2.3 mV and patients with SCI=4.8±3.6 mV, p=0.25). Mean F-wave amplitude at rest was higher in SCI (2.7±1.8% M-max) compared to healthy controls (1.2±0.7% M-max, p=0.06). We found that during 70% of MVC the mean F-wave amplitude in the TA muscle was increased (F_(1,11)_=15.7, p=0.002) to a larger extent in patients (by 60.0±60.7%) compared to healthy controls (by 21.2±28.9.0%; F_(1,11)_=2.3, p=0.04). Furthermore, F-wave persistence in the TA was increased during 70% of MVC compared to rest in healthy controls (by 15.1±25.1%) and in patients with SCI (by 34.6±35.9%; F_(1,11)_=8.6, p=0.01).

Overall, these results demonstrate impaired MEP crossed facilitation in patients with cervical SCI when the motoneurons of the muscle tested were located at or within 5 segments below the injury site during strong unilateral voluntary contractions. However, MEP crossed facilitation was preserved beyond 5 segments below the injury and aberrantly high at a distance (>15 segments) from the injury. The aberrant MEP crossed facilitation at a distance from the injury is related, at least in part, to aberrant increases in spinal cord excitability.

### MEPs above the injury site

**Figure 4:**
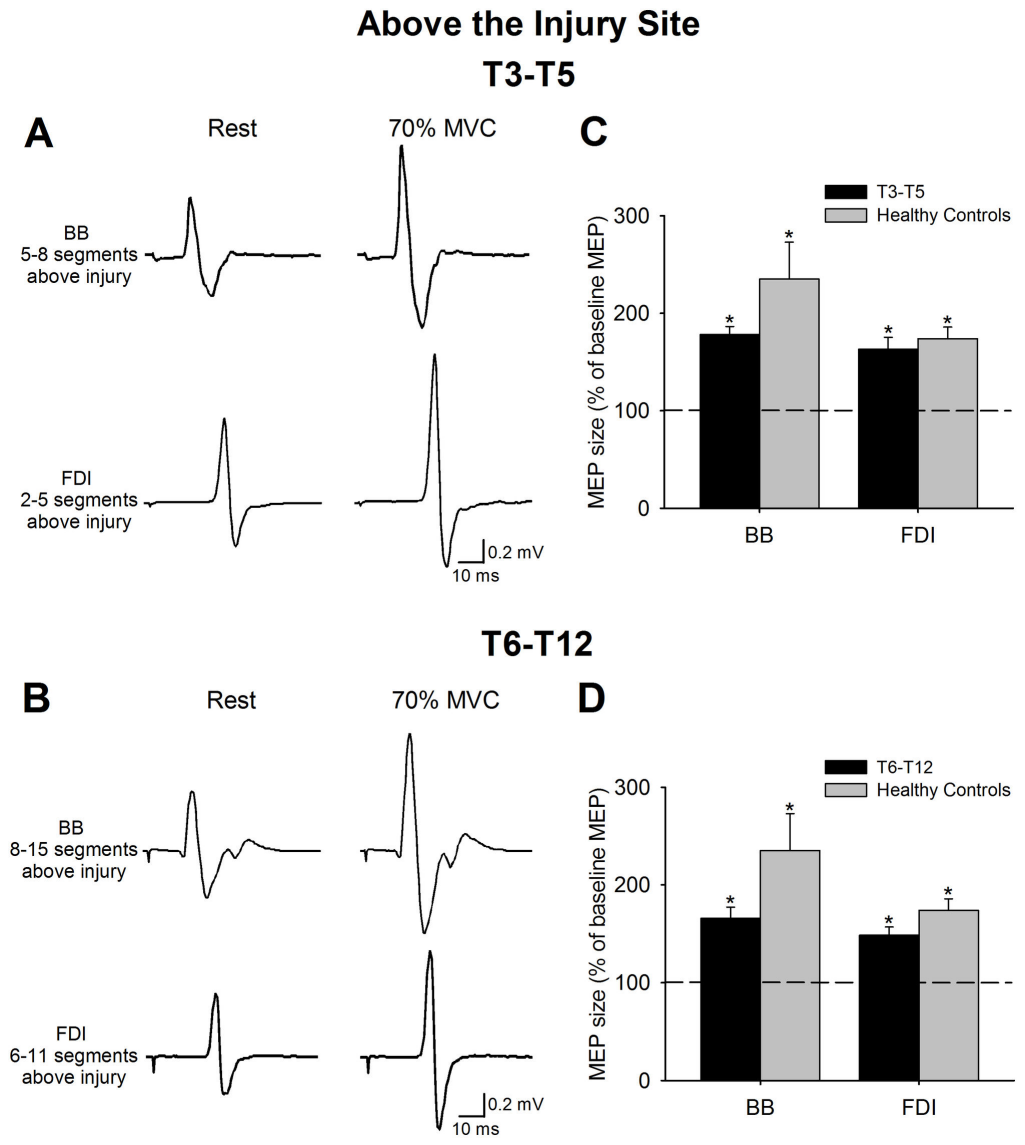
MEPs above the injury site. MEPs recorded from the resting BB and FDI of representative patients with a T3-T5 (A) and T6-T12 (B) injury, while the other side remained at rest or performed 70% of index finger abduction or elbow flexion. Group data (C, healthy controls, n=17 and T3-T5, n=6; D, healthy controls, n=17 and T6-T12, n=14). The abscissa shows the muscle tested (BB and FDI). The ordinate shows the size of FDI and BB MEPs as a % of the baseline FDI and BB MEPs. Note that the increase in FDI and BB MEP size during contralateral index finger abduction and elbow flexion in both groups of patients was similar to healthy controls. Error bars indicate SEs. *p*<*0.05.

Figure 4 illustrates MEPs from representative patients with thoracic SCI, recorded from the resting FDI and BB, during contralateral index finger abduction and elbow flexion (70% of MVC). Note that patients with injuries between T3-T5 and T6-T12 showed an increase in MEP size in the FDI and BB during 70% of MVC compared to rest. 

Repeated-measures ANOVA showed a significant effect of task (F_(1,18)_=13.6, p=0.002), but not of group (F_(2,18)_=1.9, p=0.18), or in their interaction (F_(2,18)_=1.9, p=0.18) on BB MEPs size. Indeed, BB MEP size was similarly increased during 70% of MVC compared to rest in healthy controls (235.1±125.4%, p=0.005) and in patients with T3-T5 (178.2±14.5%, p=0.01 Fig. 4AC) and T6-T12 (148.5±20.0%, p=0.001; Fig. 4BD) injuries. Furthermore, MEP size in the FDI muscle was increased during 70% of MVC compared to rest in healthy controls (174.1±48.3%, p<0.001) and in patients with T3-T5 (163.2±30.0%, p=0.004; Fig. 4AC) and T6-T12 (161.6±40.1%, p<0.001; Fig. 4BD) injuries to a similar extent (F_(2,34)_=0.4, p=0.69). Mean background rectified EMG in the resting side remained similar across conditions in both muscles (healthy controls, p=0.10; T3-T12 SCI, p=0.48). Overall, these results indicate that MEP crossed facilitation was present, and to a similar extent as in healthy controls, when the motoneurons of the muscle tested were located above the injury site regardless of the distance from the injury epicenter. 

### MEPs in all muscles and patients tested

**Figure 5:**
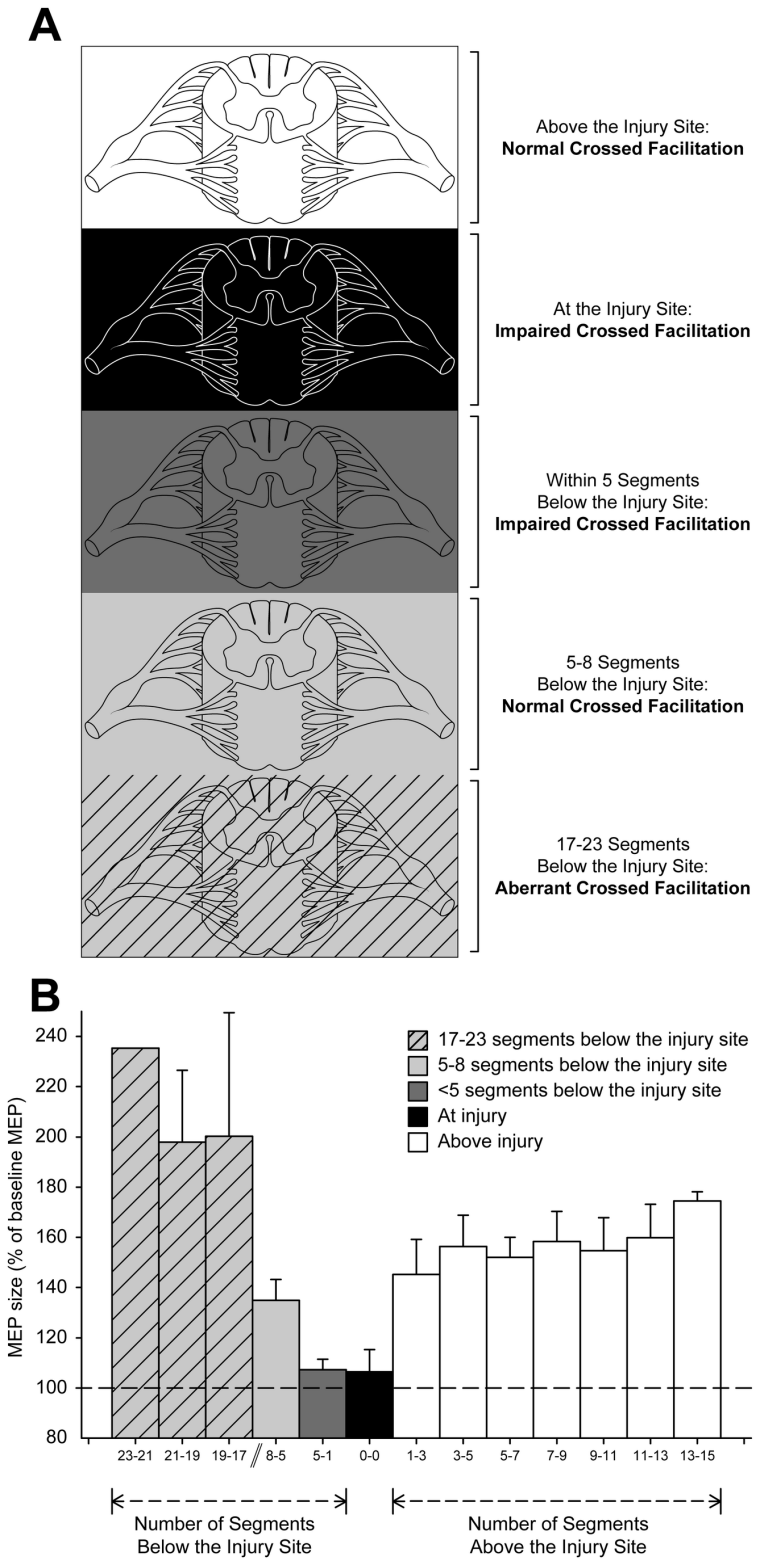
Segmental level of injury and MEPs. Schematics of the spinal cord illustrating segments above the injury (white area), at the injury site (black shaded area), within 5 segment below the injury (dark gray shaded area) and greater than 5 segments below the injury site (5-8 segments light shaded area; 17-23 segments light gray-striped area). (B) MEPs recorded from the BB, FDI, and TA in all motor tasks and patients tested are plotted as a function of the segmental level of injury. The abscissa shows the number of segments (grouped by 3 segments) from all muscles tested. The ordinate shows the size of MEPs as a % of the baseline MEPs in all muscles. Note that when the muscle tested was at or within 5 segments below the injury the size of MEPs remained unchanged during 70% of MVC compared to rest. Whereas, when the muscle tested was more than 5 segments below the injury the size of MEPs was increased and aberrantly high at longer distances during 70% of MVC compared to rest. Error bars indicate SEs.

Figure 5 illustrates the group data of MEP size (as a % of the MEP baseline) plotted against the number of segments from the injury site in all patients. MEPs in all muscles and motor tasks examined were included in the graph. Note that the magnitude of MEP size was increased when the motoneurons of the muscle tested were located above the injury site (white area, Fig. AB) during 70% of MVC compared to rest, but remained unchanged at the injury site or within 5 segments below the injury site (black and dark gray shaded areas, Fig. 5AB). More than 5 segments below the injury site, MEP size was increased during 70% of MVC compared to rest (5-8 segment; light gray shaded area, Fig. 5AB) and aberrantly increased at longer distances (17-23 segments; light gray-striped shaded area, Fig. 5AB). 

## Discussion

The present study investigated the degree of impairment in crossed corticospinal facilitation in muscles above, at, and below a SCI. We found in patients with chronic SCI that crossed corticospinal facilitation was normal in muscles above the injury, absent in muscles at or within 5 segments below the injury, and present in muscles beyond 5 segments below the injury. Crossed corticospinal facilitation was aberrantly high in muscles distant (>15 segments) from the injury and accompanied by increased motoneuronal excitability. These findings suggest that corticospinal axon degeneration does not spread rostral to the lesion, and highlight the potential of caudal regions distant from the injury to facilitate residual corticospinal output after chronic incomplete SCI.

### MEP crossed facilitation in muscles at the injury site

Our results in healthy controls are consistent with previous studies showing that a strong isometric voluntary contraction with an arm or leg muscle increases the size of MEPs in the contralateral homologus resting mucle, i.e., evoked crossed corticospinal facilitation [1–5,10,32]. In patients, we found that the increase in MEPs in muscles innervated by motoneurons located at the injury site was absent during strong voluntary contraction of the opposite limb, indicating a lack of crossed corticospinal facilitation. This result is consistent with previous studies suggesting that crossed interactions at the spinal cord level provide a mechanism for crossed corticospinal facilitation in humans [10,25]. Neuronal death and axonal damage occur at the site of injury where cystic cavities and a glial scar develop over time [12,33,34]. Axonal damage/loss in crossed networks and motoneurons at the lesion site may contribute to the lack of crossed corticospinal facilitation. Corticospinal and spinal networks cross the midline to connect with contralateral motoneurons in the gray matter [35], which is especially vulnerable because of the high degree of vascularization causing large hemorrhages after a contusive impact [36–38]. Therefore, it is possible that damage to this region abolished crossed corticospinal facilitation. However, it is less likely that significant damage/loss of spinal motoneurons was a main contributor to our findings since we showed that the size of the M-max was similar across patients with cervical SCI including those with or without preserved crossed corticospinal facilitation. 

### MEP crossed facilitation in muscles below the injury site

We found that the size of MEPs in muscles innervated by motoneurons located within 5 segments below injury site was absent during strong voluntary contraction of the opposite limb, indicating a lack of crossed corticospinal facilitation. This is in agreement with our previous results patients with chronic cervical SCI [10]. Several changes in the corticospinal tract occur in the segments below and close to the injury site that could result in the lack of or impaired crossed corticospinal transmission in the corresponding muscles. Axonal degeneration below the lesion [19,33,39,40] may lead to reduced contralateral corticospinal drive. In agreement, TMS studies showed that corticospinal drive is impaired in muscles below a SCI with delayed MEP latencies and decreased MEP amplitudes [41–44]. Importantly, we found that the size of MEPs in muscles innervated by motoneurons located beyond 5 segments caudal to the injury was increased and aberrantly high distant (>15 segments) from the injury. An intriguing question is how MEP crossed corticospinal facilitation was preserved in muscles innervated by motoneurons beyond 5 segments below the injury site. One possibility is that spontaneous sprouting and plasticity of other systems contributed to mediate these effects. Evidence has shown that extensive sprouting takes place by midline-crossing corticospinal projections below a SCI [24]. Injury-induced sprouting has been demonstrated in injured and uninjured corticospinal axons close and at a distance from the injury [12,22,45–47]. Corticospinal sprouts connecting with neurons with short projections are typically lost in time, whereas those connecting with neurons with longer projections survive [26]. This might contribute to our results of absent MEP crossed facilitation at lower segments close to the injury and present MEP crossed facilitation at more distant segments. However, even if sprouting is present a few segments below the injury these new connections may function inappropriately or not at all. In monkeys and in other animals, the degree to which corticospinal sprouts make functional connections to other structures is unknown [23]. Indeed, the remarkable increase in dendritic spine length after SCI suggests that corticospinal neurons acquire an immature pattern of synaptic connectivity [48]. This is consistent with our findings indicating that the strength of MEP crossed facilitation was more pronounced in the TA than the FDI muscle in our patients. This may represent aberrant plasticity as part of the reorganization process that occurs in spinal motoneurons after SCI. The TA motoneurons are located in a region close to the central pattern generator, which has been shown to undergo plasticity after the injury and contribute to the recovery of motor function. In agreement, our finding demonstrate a further increase in TA motoneuronal excitability during 70% of MVC compared to rest in patients than in healthy controls. An alternative explanation for the present aberrant crossed corticospinal facilitation is that intraspinal networks distant from the lesion are still intact. However, this seems less likely because tissue degeneration extends several segments below the lesion [49–51] and deficits in spinal interneurons can be seen 5 or more segments below the lesion [52].

The propriospinal network [53] could also be involved in mediating these effects. Lesions to the spinal cord can result in corticospinal sprouts which establish connections with propriospinal neurons projecting to distant spinal cord segments [22,26]. After a spinal lesion propriospinal commissural interneurons can regenerate across the lesion and form new synaptic connections with motoneurons [24]. Furthermore, reorganization at longer distances mediated by long propriospinal projections below the injury contributes to further enhance physiological and motor outcomes after SCI [27]. 

It is less likely that our results relate to the extent and configuration of the injury because the degree of sprouting in the corticospinal pathway might be influenced by the region of the corticospinal tract that was damaged [54] and our results showed a similar aberrant plasticity at longer distance in patients with C2-C4 and C5-C7 injuries. Furthermore, although possible, it is less likely that changes in the resting state of the muscles tested contributed to our results. First, we found no differences in the mean rectified background EMG activity in the resting limb in all muscles tested across conditions. Second, if spinal motoneurons were closer to their discharge threshold after injury an increase in MEP size in all muscles tested during 70% of MVC would be expected while our results were specific to the distance from the injury. 

Regardless of the mechanisms contributing to our results, or the possible additional contribution of other descending or new pathways, our findings clearly demonstrate that MEP crossed facilitation is absent at and within 5 segments below the injury, present beyond 5 segments, and aberrantly high at longer distance below the injury. The role of this increase in crossed corticospinal facilitation at these distant levels in the spinal cord remains to be elucidated. 

### MEP crossed facilitation in muscles above injury site

We found that the size of MEPs in muscles innervated by motoneurons located above the injury site was similar in patients with thoracic SCI and in healthy controls. In agreement with previous studies, we also demonstrate that measurements of corticospinal output such as MEP-max and MEP latency in patients with thoracic injuries were comparable to healthy controls [55], suggesting a low degree of reorganization in the corticospinal tract above the injury site. The extent to which corticospinal axons reorganize above the injury remains controversial [17–19]. Animal studies have shown that rostral corticospinal axons survive depending on the proximity to the injury zone [12,20], showing variable amounts of retrograde degeneration [13–15]. While some human studies indicate that degeneration in the corticospinal tract occurs above a SCI with a gradual increase in corticospinal axonal density at increasing distances [17,18], other did not report such changes [19,39]. Together, our data of intact MEP crossed facilitation and normal MEP latency and amplitudes in muscles above the injury supports the view that corticospinal degeneration rostral to the lesion does not spread to distant sites [19,39].

### Functional significance

A number of studies have noted the possibility that crossed corticospinal facilitation might benefit motor output in a more affected limb in patients with motor disorders [7,8,55–58], including SCI [9,10]. Our results indicate that this may especially be true for muscles distant from the injury. The aberrant amount of MEP crossed facilitation at distant levels below the injury might offer a new avenue to facilitate corticospinal function, although the functional role of this phenomenon remains to be determined. Our findings also suggest that the physiological information gathered from our studies may be relevant in the interpretation and design of bilateral motor training strategies in patients with SCI. We speculate that in a motor training strategy if the motoneurons of the trained muscle are located at or close to the injury site, where crossed corticospinal facilitation is impaired, benefit less from crossed interactions in the corticospinal pathway will be absent or decreased; whereas in muscles with motoneurons located at a distance from the injury site, where crossed corticospinal facilitation is present or aberrantly increased, additional inputs through facilitatory crossed interactions will be present. These physiological interactions may, to some extent, contribute to the presence, absence, or variability among effects after bilateral training of upper [59] and lower [60] limb muscles in patients with SCI. 
